# Event and Comment

**Published:** 1918-10

**Authors:** 


### DENTAL REGISTER

Vol. LXXII OCTOBER, 1918 No. 10

### EVENT AND COMMENT

**The War and Dental Education**

The world is very generally disturbed just now by the great conflict in progress in Europe, and the process of dental instruction is beginning to feel the reaction like many other peaceful processes. It has been thought best by the United States government to control directly the process of dental training and for this purpose it has commandeered the colleges and prescribed the methods of conducting the dental curriculum as well as that of all other educational institutions where male students are being educated. It is not the purpose of the government to change the plans of instruction materially, but for the most part in a supervisory way only. The object being to focus the aims of the course of instruction on the preparation of graduates who can meet the requirements of military dentistry in the most capable manner, and in the shortest time consistent with thoroughness. At the present date there is no shortage of dentists in the Army needs but should the war continue for many years it is probable there will be an increasing need for a high grade of dentistry in connection with the several departments of service. At the present stage of this interruption of the traditional forms of imparting dental instruction no one can predict the consequences of this interruption, and we have no disposition to question the necessity for the move. Every college seems willing to accept the challenge to be of service, with good grace, and they are exhibiting a spirit of determination to do their part. We are confident that whatever part the dental colleges are called upon to take they will do the best they can to meet the expectations of the government. Like every other preparatory war work we shall undoubtedly make some mistakes and we may not measure up to the task as completely as some may desire. If the war shall continue long enough we shall do better work as time passes, and possibly we shall learn some things that will be of greater value in the preparation of students for future civil practice than any losses we may sustain by the interruption. Whether we shall gain or lose by the change is a matter of comparatively small concern when viewed along with the importance of the great crisis which requires the sacrifice. It is our opportunity to contribute our part and it is fine to see how eagerly and contentedly all hands are entering into the spirit of the effort to help win the war. With a spirit of helpful co-operation backing up this movement we may confidently expect a contribution to the cause that shall be most helpful, and a reaction that shall place the work of dental education on a more uniform and standardized basis in the future. The weak colleges will drop out and the better ones will develop strength and character because of the experience through which they will be guided by the strong arm of the government. While the times in which we are living are full of inconveniences and dreadful events, it certainly is grand to be living and have a chance to lend a hand in a great revolution which may usher in a new and better civilization than we have ever known.

**Stenographers and Typists** The United States Civil Service Commission is sending out a call for stenographers and typewriters. It seems impossible to secure enough help of this kind to carry on the great and constantly increasing demands for this kind of clerical help. The government pays good wages and is erecting immense buildings for their use. Although living accommodations in Washington are scarce and expensive, carefully inspected rooming and boarding houses are to be had through government agencies, at $40 per month. The government is also building residence halls for women. This is a good way in which our women can do their bit for carrying on the war, and anyone having such talent will find this service profitable and agreeable. If any of our readers can pass this word on to such as could qualify for either kinds of work they will also help our war work effectively.

**National War Council of the Y. M. C. A.** The week of November 11 to 18 is to be given over to a great "drive" to raise money to help carry on the Christian work among our soldiers. In this drive all organizations doing similar work are organized to economize efforts in solicitation, etc. The Y. M. C. A., Y. W. C. A., Catholic War Council, Jewish Welfare Board, War Community Service, American Library Association and Salvation Army, have united and each organization will have its share of all the contributions. There can be no question as to the value of these various services and of their singleness of purpose and it is a grand thing to find all these diverse interests consolidating under one big management in soliciting their funds. The campaign will not lack for enthusiasm, and the full amount of money needed should be forthcoming. We trust all our readers may accept the word of General Pershing and the evidence given in letters from the soldiers themselves, as to the merits of the work done by these organizations. The Y. M. C. A. and like organizations have brought cheer, as well as creature comforts, to thousands who otherwise would have had no contact with any of the higher things than the terrible scenes of actual war. Let us all keep constantly before our brave fighting men the wholesome Christian ideals that shall prevent them from falling into some of the terrible atrocities about which we have heard so much and by which they may be tempted to do similar deeds.

